# Which intervention is better for malaria vector control: insecticide mixture long-lasting insecticidal nets or standard pyrethroid nets combined with indoor residual spraying?

**DOI:** 10.1186/s12936-017-1987-5

**Published:** 2017-08-16

**Authors:** Corine Ngufor, Josias Fagbohoun, Jessica Critchley, Raphael N’Guessan, Damien Todjinou, David Malone, Martin Akogbeto, Mark Rowland

**Affiliations:** 1 London School of Hygiene and Tropical Medicine (LSHTM), London, UK; 2 Centre de Recherches Entomologiques de Cotonou (CREC), Cotonou, Benin; 3 Pan African Malaria Vector Research Consortium (PAMVERC), Cotonou, Benin; 4 Innovative Vector Control Consortium (IVCC), Liverpool, UK

**Keywords:** Experimental huts, Chlorfenapyr, Interceptor^®^ G2 LN, Combined interventions, Mixture LLIN, Pyrethroid resistance, Long-lasting insecticidal nets, Indoor residual spraying, *Anopheles*, Cove Benin

## Abstract

**Background:**

Malaria control today is threatened by widespread insecticide resistance in vector populations. The World Health Organization (WHO) recommends the use of a mixture of unrelated insecticides for indoor residual spraying (IRS) and long-lasting insecticidal nets (LNs) or as a combination of interventions for improved vector control and insecticide resistance management. Studies investigating the efficacy of these different strategies are necessary.

**Methods:**

The efficacy of Interceptor^®^ G2 LN, a newly developed LN treated with a mixture of chlorfenapyr (a pyrrole) and alpha-cypermethrin (a pyrethroid), was compared to a combined chlorfenapyr IRS and Interceptor^®^ LN (a standard alpha-cypermethrin LN) intervention in experimental huts in Cove Southern Benin, against wild, free-flying, pyrethroid-resistant *Anopheles gambiae s.l.* A direct comparison was also made with a pyrethroid-only net (Interceptor^®^ LN) alone and chorfenapyr IRS alone.

**Results:**

WHO resistance bioassays performed during the trial demonstrated a pyrethroid resistance frequency of >90% in the wild *An. gambiae s.l.* from the Cove hut site. Mortality in the control (untreated net) hut was 5%. Mortality with Interceptor^®^ LN (24%) was lower than with chlorfenapyr IRS alone (59%, P < 0.001). The combined Interceptor^®^ LN and chlorfenapyr IRS intervention and the mixture net (Interceptor^®^ G2 LN) provided significantly higher mortality rates (73 and 76%, respectively) and these did not differ significantly between both treatments (P = 0.15). Interceptor LN induced 46% blood-feeding inhibition compared to the control untreated net, while chlorfenapyr IRS alone provided none. Both mixture/combination strategies also induced substantial levels of blood-feeding inhibition (38% with combined interventions and 30% with Interceptor^®^ G2 LN). A similar trend of improved mortality of pyrethroid-resistant *An. gambiae s.l.* from Cove was observed with Interceptor^®^ G2 LN (79%) compared to Interceptor LN (42%, P < 0.001) in WHO tunnel tests.

**Conclusion:**

The use of chlorfenapyr and alpha-cypermethrin together as a mixture on nets (Interceptor^®^ G2 LN) or a combined chlorfenapyr IRS and pyrethroid LN intervention provides improved control of pyrethroid-resistant malaria vectors by inducing significantly higher levels of mortality through the chlorfenapyr component and providing personal protection through the pyrethroid component. Both strategies are comparable in their potential to improve the control of malaria transmitted by pyrethroid resistant mosquito vectors.

**Electronic supplementary material:**

The online version of this article (doi:10.1186/s12936-017-1987-5) contains supplementary material, which is available to authorized users.

## Background

Indoor residual spraying (IRS) and long-lasting insecticidal nets (LNs) have contributed immensely to recent reductions in malaria burden [1]. The sustainability of this impact is however threatened by widespread resistance to the insecticides delivered through these interventions, especially pyrethroids [2]. Modelling studies have suggested that if nothing is done about insecticide resistance, the recent fragile gains in malaria control could be reversed, thus calling for urgent concerted efforts to mitigate these threats [2].

Pyrethroids are currently the only insecticide used on WHOPES-recommended LNs owing to their efficacy, safety, low cost, and excito-repellent effect on mosquitoes, an insecticidal property which is essential for preventing mosquito biting [3]. The identification of alternative insecticides for treating LNs has been very challenging because most non-pyrethroid insecticides lack this excito-repellent effect which is responsible for providing personal protection to net users and increasing acceptability of the intervention [4, 5]. As an interim solution, novel non-pyrethroid insecticides being developed for vector control can be mixed with pyrethroids on bed nets or deployed as IRS in combination with pyrethroid LNs. By presenting the non-pyrethroid insecticide together with a pyrethroid, whether as a mixture on the net or a combined intervention, there is potential to maintain personal protection through the pyrethroid component while achieving high mosquito mortality rates through the non-pyrethroid. The use of unrelated insecticides in mixtures or as a combined intervention for malaria vector control is recommended by the Global Plan for Insecticide Resistance Management (GPIRM) because it also has the potential to preserve insecticide susceptibility in areas where resistance to both active ingredients is still rare [2, 6].

With increased funding for vector control, especially from the US President’s Malaria Initiative (PMI), some vector control programmes have deployed non-pyrethroid IRS together with pyrethroid LNs with the objective of improving malaria control [7, 8]. While the impact of this approach has been controversial, some studies have shown that improved vector control is possible but may depend on the insecticide being used for IRS and the resistance status and behaviour of the target vector population [9, 10]. By using mixtures of non-pyrethroid insecticides with pyrethroids on LNs it may also be possible to achieve improved vector control with a single intervention since the underlying concept is similar to the combined non-pyrethroid IRS and pyrethroid LN intervention. To provide some guidance to product development companies involved in the development of novel public health insecticides and vector control programmes, studies comparing the efficacy of both approaches are necessary.

Chlorfenapyr is a novel pyrrole insecticide, which has shown potential to significantly improve the control of pyrethroid resistant malaria vectors [4, 11–14]. It shows no cross-resistance to current public health insecticides and has thus been evaluated for IRS and mosquito nets [14]. However, because chlorfenapyr lacks excito-repellency, it confers very limited personal protection to the user when applied alone [12]. Chlorfenapyr can therefore be mixed with pyrethroids on LNs and for IRS or deployed as IRS in combination with pyrethroid LNs with the aim of improving mortality while providing personal protection through the excito-repellent property of the pyrethroid. In this study, the efficacy of Interceptor^®^ G2 LN, a newly developed mixture LN treated with chlorfenapyr and alpha-cypermethrin (a pyrethroid) was compared to a combined chlorfenapyr IRS and pyrethroid-only LN (Interceptor^®^ LN) intervention in experimental huts against pyrethroid-resistant *Anopheles gambiae* sensu lato (*s.l*.) in Cove, Southern Benin.

## Methods

### Study site and experimental huts

The study was performed in an experimental hut station situated at the centre of a large rice-growing field in Cove, Southern Benin. The rice paddies provide extensive breeding sites for *An. gambiae s.l.* throughout the year. The rainy season extends from March to October and the dry season from November to February. The trial was performed between June and September 2015 in five experimental huts of the West African design. Preliminary studies revealed that the huts were equally attractive to mosquitoes. The experimental huts are built on concrete plinths surrounded by water-filled moats to prevent entry of scavenging ants, and have veranda traps to capture the exiting mosquitoes. The walls are made of brick plastered with cement on the inside, with a corrugated iron roof. The huts have a ceiling of palm thatch and four window slits (1-cm gap) on the walls through which mosquitoes enter. The local vector population in Cove is resistant to pyrethroids and DDT and consists of a mixture of *Anopheles coluzzii* and *An. gambiae* sensu stricto (*s.s.*), with the latter occurring at lower proportions (23%) and only in the dry season [15]. Molecular analysis revealed a L1014F kdr allele frequency of 89%. Micro-array studies performed a year before the study also found CYP6P3, a P450 validated as an efficient metabolizer of pyrethroids [16] to be overexpressed in Cove [15].

### WHO susceptibility bioassays

To determine the frequency of resistance to pyrethroids and organochlorines in the wild *An. gambiae* Cove vector population during the trial, mosquitoes that emerged from larvae collected from breeding sites at the experimental hut station were tested in WHO cylinder bioassays treated with permethrin 0.75%, deltamethrin 0.05% and DDT 4%. Comparison was made with the laboratory-maintained susceptible *An. gambiae* Kisumu strain. A total of ~100 mosquitoes were exposed in batches of 25 for 1 h to each insecticide and the control and deaths were scored 24 h later.

### Experimental hut treatments

The following treatments were tested in the experimental huts:

1. Control (untreated net).
2. Interceptor^®^ LN (alpha-cypermethrin—200 mg/sq m treated LN).
3. Chlorfenapyr (BASF Phantom 21.45% SC) IRS applied at 250 mg/sq m.
4. Chlorfenapyr IRS applied at 250 mg/sq m + Interceptor^®^ LN.
5. Interceptor^®^ G2 LN (chlorfenapyr 200 mg/sq m + alpha-cypermethrin 100 mg/sq m mixture LN).

Chlorfenapyr IRS was applied using a Hudson Xpert compression sprayer equipped with a 8002 flat fan nozzle. The palm thatch used on the ceiling of the hut was sprayed lying flat on the floor (outside the hut) and allowed to air dry for 1–2 h before being fitted to the ceiling of the hut. To estimate the quality of the spray applications, the insecticide solution left in the spray tank after spraying each hut was poured into a measuring cylinder to determine the volume sprayed. The actual sprayed volume was within 10% of the target insecticide volume required for each of the chlorfenapyr IRS treated huts, suggesting that the spraying was accurate.

All mosquito nets used in the study were unwashed. To simulate wear and tear during field use, the nets were intentionally holed with 6 holes of area 16 sq cm (2 holes on each side and 1 hole on each end) following WHO guidelines [17]. To reduce bias due to hut position, the mosquito nets were rotated between the respective net treatments arms on a weekly basis. Three nets were prepared per treatment arm and these nets were rotated every 2 days within each week of the trial.

### Trial procedure

The trial ran for 54 nights between June and September of 2015. Human volunteer sleepers slept in the huts from 21:00 to 05:00 each night and were rotated through the huts daily to account for individual attractiveness to mosquitoes. At dawn, the volunteer sleepers collected dead mosquitoes in the room of the hut and under the bed nets and all mosquitoes that escaped into the veranda, using torches and aspirators. The mosquitoes were then transferred to the laboratory for processing where they were identified and scored for their blood-feeding status, mortality and hut position. Delayed mortality was recorded every 24 h up to 72 h, to account for the slow-acting effect of chlorfenapyr. Mosquitoes were held at 25 ± 2 °C during the observations.

### Outcome measures

The following outcome measures were used to assess the efficacy of the treatments in the experimental huts:

1. Deterrence: the proportional reduction in number of mosquitoes entering treated huts compared to the control hut.
2. Insecticide-induced exiting rates estimated from the proportions of mosquitoes collected from the verandas of treatment and control huts.
3. Mortality: the proportion of mosquitoes killed (immediate plus delayed) relative to the total collected.
4. Blood-feeding inhibition: the proportional reduction in blood feeding in huts with insecticide treatments relative to the untreated control.
5. Personal protection: the reduction in mosquito biting by LNs relative to untreated nets, as derived from the formula.
  $$\% \;personal\;protection\; = \;100\;\frac{(B_{u} \; - \;B_{t} )}{B_{u} }$$
  where B_u_ is the total number of blood-fed mosquitoes in the hut with the control, and B_t_ is the total number blood fed in the huts with treatment.
6. The overall killing effect of a treatment relative to the number of mosquitoes that would ordinarily enter an untreated control hut was estimated by using the following formula and expressed as a percentage:
  $$Overall \, killing \, effect \, \left( \% \right) \, = \, 100 \, \left( {K_{t} {-} \, K_{u} } \right)/T_{u}$$
  where K_t_ is the number killed in the treated hut, K_u_ is the number dying in the untreated control hut, and T_u_ is the total number collected from the control hut.

### Tunnel tests

To help explain the findings in the experimental huts, tunnel tests were performed with net samples of Interceptor LN G2 and Interceptor LN using F1 adult mosquitoes of the pyrethroid-resistant *An. gambiae s.l.* population from Cove. Comparison was made to a control net sample. Two-hundred 5–8 days old F1 female mosquitoes were exposed to each netting sample in 2 replicate tunnels. The tunnel test consists of a square glass cylinder (25 cm high, 25 cm wide, 60 cm in length) divided into two sections by means of a netting frame fitted into a slot across the tunnel. In one of the sections, a guinea pig was housed unconstrained in a small cage, and in the other Section 50 unfed female mosquitoes aged 5–8 days were released at dusk and left overnight. The net samples were deliberately holed with nine 1-cm holes to give opportunity for mosquitoes to penetrate into the animal-baited chamber for a blood meal; an untreated net sample served as the control. The tunnels were kept overnight in a dark room at 25–27 °C and 75–85% relative humidity. The next morning, the numbers of mosquitoes found alive or dead, fed or unfed, in each section were scored. Live mosquitoes were provided with 10% glucose solution and delayed mortality recorded after 72 h.

### Data analysis

Proportional outcomes (blood feeding, exiting and mortality) related to each experimental hut treatment were assessed using binomial generalized linear mixed models (GLMMs) with a logit link function, fitted using the ‘lme4’ package for R (version 2.15.0). A separate model was fitted for each outcome. In addition to the fixed effect of each treatment, each model included random effects to account for the following sources of variation: between the huts, between the sleepers, between the weeks of the trial, and finally, an observation-level random effect to account for variation not explained by the other terms in the model (overdispersion).

Differences in deterrence, overall killing effect and personal protection between the treatments were analysed using negative binomial regression based on numbers entering and numbers blood fed and killed, respectively, with adjustment for the abovementioned covariates.

## Results

The WHO susceptibility tests performed on F1 adults *An. gambiae s.l.* mosquitoes collected as larvae from breeding sites near in the experimental hut station in Cove showed high survival rates (>90%) after exposure to pyrethroids (permethrin 0.75% and deltamethrin 0.05%) and organochlorine DDT-treated papers thus confirming the high levels of resistance in the Cove vector population to these insecticides. Mortality with the susceptible *An. gambiae* Kisumu strain was 100% with all three insecticides. No mortality was recorded in the control. Detailed results on the susceptibility bioassays are provided in Additional file 1: Table S1.

### Experimental hut trial results

**Fig. 1:**
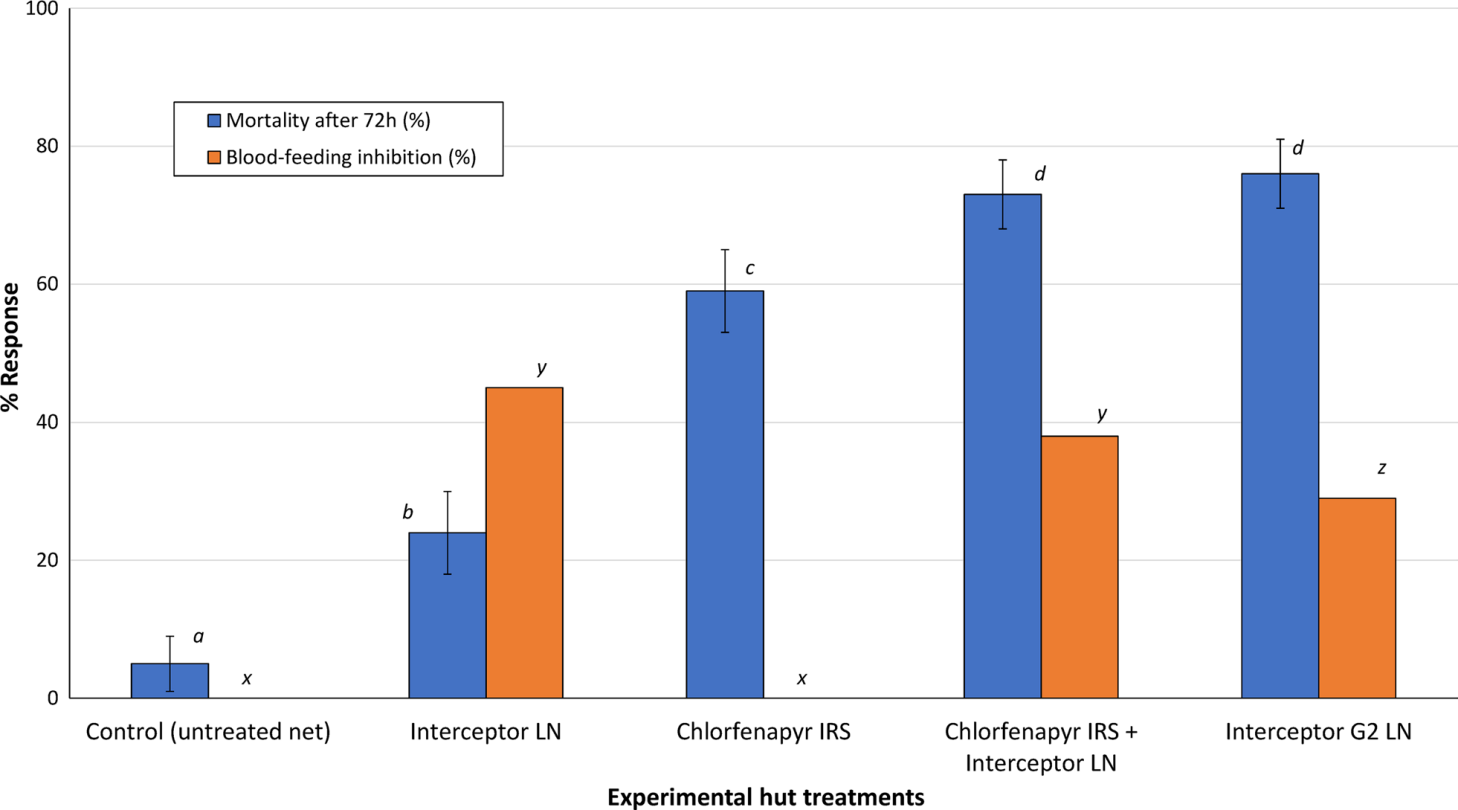
Mortality (%) and blood-feeding inhibition (%) of pyrethroid-resistant *Anopheles gambiae* in experimental huts in Cove, Benin. The *blue bars* represent mortality rates while the *orange bars* represent blood-feeding inhibition rates relative to the control. *Bars of the same colour bearing the same letter* label are not significantly different at the 5% level (P < 0.05, GLMM). *Error bars* represents 95% CIs. Interceptor^®^ G2 LN is a new mixture LN treated with chlorfenapyr and alpha-cypermethrin while Interceptor^®^ LN is a standard approved LN treated only with alpha-cypermethrin

The experimental hut trial results are presented in Tables 1, 2, 3 and Fig. 1. A total of 1153 female *An. gambiae s.l.* were collected in the experimental huts during the trial. Deterrency with Interceptor^®^ LN (44%) was higher than with Interceptor^®^ G2 LN (19%) and this did not change significantly compared to the combined chlorfenapyr IRS and Interceptor^®^ LN hut (49%). Chlorfenapyr IRS alone induced 16% deterrence, however, because the IRS treatments could not be all rotated between the huts, it was not possible to attribute this effect entirely to the treatment.

**Table 1:** Entry rates of wild *An. gambiae* sl in experimental huts in Cove, Benin

	Control (untreated net)	Interceptor^®^ LN	Chlorfenapyr IRS	Chlorfenapyr IRS + Interceptor^®^ LN	Interceptor^®^ G2 LN
Total females caught	310	175	259	158	251
Average catch per night	5.7	3.2	4.8	2.9	4.6
% deterrence	–	44	16	49	19

**Table 2:** Blood feeding rates of wild pyrethroid resistant *An gambiae* sl and personal protection in experimental huts in Cove, Benin

	Control (untreated net)	Interceptor^®^ LN	Chlorfenapyr IRS	Chlorfenapyr IRS + Interceptor^®^ LN	Interceptor^®^ G2 LN
Total blood fed	210	65	209	66	120
% blood fed*	68^a^	37^b^	81^c^	42^bd^	48^d^
95% conf. interval	62–73	30–44	76–85	34–50	42–54
% blood-feeding inhibition	–	46	–	38	30
% inside net*	52^a^	26^b^	–	27^b^	32^b^
95% conf. interval	46–58	22–30	–	20–34	26–38
% personal protection	–	69	0	69	43

* Values in the same row sharing a letter superscript do not differ significantly (P > 0.05, GLMM)

**Table 3:** Overall killing effect of wild pyrethroid resistant *An gambiae* sl in experimental huts in Cove, Benin

	Control (untreated net)	Interceptor^®^ LN	Chlorfenapyr IRS	Chlorfenapyr IRS + Interceptor^®^ LN	Interceptor^®^ G2 LN
N dead after 24 h	8	20	92	57	137
N dead after 72 h	16	45	153	112	185
% corrected 24 h Mortality*	0	8^a^	34^b^	34^b^	54^c^
% corrected 72 h Mortality*	0	22^a^	57^b^	70^c^	73^c^
95% conf. interval	–	16–24	53–60	67–73	70–77
% overall killing effect	–	9	44	31	55

* Values in the same row sharing a letter superscript do not differ significantly (P > 0.05, GLMM)

### Blood feeding and personal protection

The results on blood feeding and personal protection are presented on Table 2. Blood-feeding rates with the control (untreated net) was 68%. Blood-feeding rates were lower with Interceptor^®^ LN compared to the control (68 vs 37%, P < 0.001) but did not differ significantly from the combined chlorfenapyr IRS and Interceptor^®^ LN treatment (37 vs 42% P = 0.396). The proportions fed in the huts with Interceptor^®^ G2 LN (48%) and the combined chlorfenapyr IRS and Interceptor^®^ LN treatment (42%) were also similar (P = 0.236). As expected with IRS treatments, blood-feeding rates were very high with chlorfenapyr IRS alone (81%), hence there was no evidence of blood-feeding inhibition (Fig. 1) or personal protection (Table 2) with this treatment. Blood-feeding inhibition and personal protection with Interceptor^®^ LN alone were 46 and 69%, respectively, and this did not change when the net was combined with chlorfenapyr IRS (38 and 69%, respectively, P > 0.05). Interceptor^®^ G2 LN also provided substantial levels of blood-feeidng inhibition and personal protection (30 and 43%, respectively) but these were lower than was observed with Interceptor^®^ LN and the combined intervention. Significantly smaller proportions of mosquitoes were also collected inside Interceptor^®^ LN (26–27%) and Interceptor^®^ G2 LN (32%) compared to the control (52%, P < 0.001).

### Mortality rates

Mortality results are presented in Table 3 and Fig. 1. Mortality rate with the control was 5%. Mortality with Interceptor^®^ LN was 24%. Mortality was higher with chlorfenapyr IRS alone (59%) than Interceptor^®^ LN (P < 0.001), but significantly lower compared to the combined chlorfenapyr IRS and Interceptor^®^ LN intervention (59 vs 73%, P = 0.041) (Fig. 1). The highest mortality was achieved with Interceptor^®^ G2 LN (76%) but this did not differ significantly from the combined chlorfenapyr IRS (76 vs 73%, P = 0.15). Overall killing effect was very low with Interceptor^®^ LN (9%) (Table 3). Interceptor^®^ G2 LN induced the highest overall killing effect (55%) compared to chlorfenapyr IRS alone (44%) and the combined chlorfenapyr IRS and Interceptor^®^ LN treatment (31%) (P < 0.05).

### Tunnel tests results

**Fig. 2:**
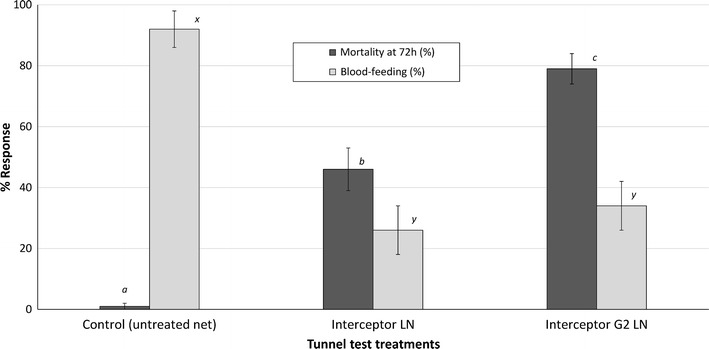
Mortality (%) and blood-feeding rates (%) of F1 adults of pyrethroid-resistant *Anopheles gambiae s.l.* from Cove in WHO tunnel tests. The *dark shade bars* represent mortality rates while the *lighter shade bars* represent blood-feeding rates. *Bars of the same colour bearing the same letter* label are not significantly different at the 5% level. *Error bars* represents 95% CIs. Interceptor^®^ G2 LN out-performed Interceptor^®^ LN in the tunnel tests

The tunnel test results with pyrethroid-resistant *An. gambiae s.l.* from Cove are presented in Fig. 2. The trend was similar to that observed in the experimental hut trial; Interceptor^®^ G2 LN out-performed Interceptor LN. Mortality in the tunnel tests was 1% with the control net and 46% with Interceptor^®^ LN. Mortality was significantly higher with Interceptor^®^ G2 LN (79%, P < 0.05). Blood feeding in the control was 92%. Blood feeding was much lower with Interceptor LN (26%) and Interceptor^®^ G2 LN (34%) but did not differ significantly between both LN types (P > 0.05). As observed in the hut trial, although blood-feeding inhibition in tunnels with Interceptor^®^ G2 LN was substantial (63%), it was lower than was achieved with Interceptor^®^ LN (72%).

## Discussion

The purpose of this study was to assess the efficacy of deploying a combination of unrelated insecticides against pyrethroid-resistant populations of malaria vectors either as a mixture on LNs or as a combined non-pyrethroid IRS and pyrethroid LN intervention. Interceptor^®^ G2 LN, a newly developed LN treated with a mixture of chlorfenapyr and alphacypermethrin was compared to a combined chlorfenapyr IRS and pyrethroid LN intervention in experimental huts in a pyrethroid-resistant area in Southern Benin. The low mortality rates (24%) achieved with the Interceptor^®^ LN (pyrethroid-only LN) is very typical of most experimental hut studies in pyrethroid-resistant areas [11, 18, 19]. However, Interceptor LN also induced substantial blood-feeding inhibition rates and personal protection demonstrating that the pyrethroid treatment on the net continues to provide some protection to the user against this high pyrethroid-resistant vector population.

By presenting two unrelated insecticides (alphacypermethrin and chlorfenapyr) to the vector population at the same time, whether as a combined chlorfenapyr IRS and alpha-cypermethrin LN approach or as a mixture on a net (Interceptor^®^ G2 LN), it was possible to significantly improve vector mortality and provide personal protection to the sleeper at the same time. Improved mortality was due to the chlorfenapyr component while personal protection was mostly due to the excito-repellent effect of pyrethroid. These mixture/combination approaches therefore show potential to restore malaria vector control in areas where the efficacy of pyrethroid LNs is being compromised by high pyrethroid resistance, to levels achievable with susceptible vector populations [20].

Although the mortality rates observed with both mixture/combination strategies were similar, the mixture net induced higher levels of overall killing effect while the combined intervention provided higher levels of personal protection. The latter was probably due to the higher concentration of alphacypermethrin in Interceptor LN compared to Interceptor^®^ G2 LN. Overall, the results revealed that the combined intervention and mixture net approaches are broadly comparable in their impact on pyrethroid-resistant malaria vector populations under field situations.

Of all the mixture-based strategies recommended by the GPIRM for mitigating insecticide resistance in malaria vectors, the combined use of non-pyrethroid IRS and pyrethroid LNs together has been more widely implemented [7, 10, 21, 22]. While some community randomized trials have failed to clearly demonstrate added protection with the combined intervention approach [21], probably due to issues related to study design, modelling studies have shown that improved impact is possible but may depend on several factors, including the type of IRS insecticide used, the residual efficacy of the IRS, the level of resistance in the targeted vector population, and the behaviour of the local vector species [22]. The improved levels of mortality and substantial personal protection obtained with the combined chlorfenapyr IRS and pyrethroid LN approach confirm previous findings [11] and demonstrate that chlorfenapyr could be a suitable IRS insecticide to complement pyrethroid LNs in high pyrethroid-resistant areas with existing high pyrethroid LN coverage. The residual protective excito-repellent effect of the pyrethroid-only net (Interceptor^®^ LN) against the wild pyrethroid-resistant malaria vectors was not confounded by the chlorfenapyr IRS treatment on the wall when combined. Improved mortality in the combined intervention compared to the chlorfenapyr IRS alone or pyrethroid LN alone may be due to the to-and-fro movement of frustrated unfed mosquitoes between the LN and the treated wall leading to increased pick up of insecticide. It could also be simply due to an additive effect of the active ingredients in both interventions when applied together. Studies evaluating the impact of combining chlorfenapyr IRS with pyrethroid LNs on malaria in community randomized trials in village clusters are also necessary.

Compared to IRS interventions, which usually require complex operational delivery systems, LNs are much easier to deliver even in the most remote communities. While the use of two active ingredients on a bed net will probably increase the total cost of manufacturing of the mixture LNs compared to the pyrethroid-only net, the operational resources required to deploy the mixture LN alone could be less demanding for control programmes in endemic resource-limited settings than deploying IRS and LNs together. Interceptor^®^ G2 LN could therefore be a more convenient and scalable vector control tool for control programmes in resource-limited settings that are faced with high pyrethroid resistance compared to the combined intervention approach.

Current criteria for efficacy claims of such novel mixture nets defined by the Vector Control Advisory Group (VCAG) of the WHO stipulate that the nets should induce >25% increase in mortality compared to a standard pyrethroid net against a vector population with >10-fold pyrethroid resistance compared to a suitable susceptible strain [23]. By outperforming Interceptor® LN with a 65% increase in mortality against the Cove vector population, which demonstrates >200-fold pyrethroid resistance [15], Interceptor® G2 LN therefore meets WHO criteria for efficacy against pyrethroid-resistant malaria vectors. These findings confirm results from recent studies with Interceptor® G2 LN which also demonstrated significantly improved mortality of pyrethroid-resistant malaria vectors compared to Interceptor® LN [24]. Further studies investigating the added impact of Interceptor® G2 LN on malaria control in community randomized trials in pyrethroid-resistant areas need to be performed.

Although improved vector control was achieved with the mixture net and combined intervention approach, one rationale for such strategies is to manage resistance by preventing further selection of insecticide-resistant genotypes. Resistance management potential should ideally be assessed in more complex, carefully designed, community randomized trials which study the impact of the interventions on resistance gene frequencies over time. Based on modelling studies, the mixture net and combined intervention strategies if applied in areas with low pyrethroid resistance could delay the emergence of resistance to chlorfenapyr, thus increasing the useful life of the insecticide [6]; a hypothesis worth investigating.

## Conclusions

Interceptor^®^ G2 LN, a mixture net treated with alpha-cypermethrin and chlorfenapyr and the combined use of chlorfenapyr IRS and Interceptor^®^ LN provided comparable levels of improved control of pyrethroid-resistant malaria vectors. Given that Interceptor^®^ G2 LN is long lasting and much easier to deploy, the mixture net could be a more reliable and scale-able means for improving the control of pyrethroid-resistant malaria vectors compared to the combined intervention approach. Further studies comparing the added impact on malaria control, cost-effectiveness and resistance management potential of these two strategies in community randomized trials are necessary.

## Additional file

**Additional file 1: Table S1.** WHO susceptibility bioassay results with pyrethroid resistant *An gambiae* sl from Cove, Benin.Performed June–August 2015.

## Abbreviations

LN: long-lasting insecticidal nets
IRS: indoor residual spraying
WHO: World Health Organisation
VCAG: Vector Control Advisory Group
GPIRM: Global Plan for Insecticide Resistance Management
WHOPES: WHO Pesticide Evaluation Scheme
