# Supplementary material for: Which intervention is better for malaria vector control: insecticide mixture long-lasting insecticidal nets or standard pyrethroid nets combined with indoor residual spraying?
Source: Malar J. 2017 Aug 16;16:340. doi: 10.1186/s12936-017-1987-5 (PMC5559808; doi:10.1186/s12936-017-1987-5)
Supplement: Supplementary file 1 — Additional file 1: Table S1. WHO susceptibility bioassay results with pyrethroid resistant An gambiae sl from Cove, Benin.Performed June–August 2015. [file 12936_2017_1987_MOESM1_ESM.docx]

|  | **Insecticide** | **N exposed** | **N knocked down at 1h** | **% Knock down** | **N dead at 24h** | **% Mortality at 24h** |
| --- | --- | --- | --- | --- | --- | --- |
| *An gambiae* Kisumu | Control | 120 | 0 | 0 | 0 | 0 |
|  | Permethrin 0.75% | 99 | 97 | 98 | 99 | 100 |
|  | Deltamethrin 0.05% | 107 | 104 | 97 | 107 | 100 |
|  | DDT 4% | 103 | 88 | 85 | 103 | 100 |
| *An gambiae* sl Cove | Control | 115 | 0 | 0 | 0 | 0 |
|  | Permethrin 0.75% | 106 | 1 | 1 | 8 | 8 |
|  | Deltamethrin 0.05% | 103 | 15 | 15 | 10 | 10 |
|  | DDT 4% | 112 | 2 | 2 | 9 | 8 |

**Table S1: WHO susceptibility bioassay results with pyrethroid resistant *An gambiae* sl from Cove, Benin – Performed June – August 2015**
